# Genetic and environmental factors in interstitial lung diseases: current and future perspectives on early diagnosis of high-risk cohorts

**DOI:** 10.3389/fmed.2023.1232655

**Published:** 2023-08-03

**Authors:** Stefan Cristian Stanel, Jack Callum, Pilar Rivera-Ortega

**Affiliations:** ^1^Interstitial Lung Disease Unit, Wythenshawe Hospital, Manchester University NHS Foundation Trust, Manchester, United Kingdom; ^2^Faculty of Biology, Medicine and Health, University of Manchester, Manchester, United Kingdom

**Keywords:** familial pulmonary fibrosis, familial interstitial pneumonia, telomere shortening, interstitial lung disease, environment, genetics, FPF, FIP

## Abstract

Within the wide scope of interstitial lung diseases (ILDs), familial pulmonary fibrosis (FPF) is being increasingly recognized as a specific entity, with earlier onset, faster progression, and suboptimal responses to immunosuppression. FPF is linked to heritable pathogenic variants in telomere-related genes (TRGs), surfactant-related genes (SRGs), telomere shortening (TS), and early cellular senescence. Telomere abnormalities have also been identified in some sporadic cases of fibrotic ILD. Air pollution and other environmental exposures carry additive risk to genetic predisposition in pulmonary fibrosis. We provide a perspective on how these features impact on screening strategies for relatives of FPF patients, interstitial lung abnormalities, ILD multi-disciplinary team (MDT) discussion, and disparities and barriers to genomic testing. We also describe our experience with establishing a familial interstitial pneumonia (FIP) clinic and provide guidance on how to identify patients with telomere dysfunction who would benefit most from genomic testing.

## 1. Introduction

The scope of interstitial lung diseases (ILDs) is extremely wide (over 200 disorders). The most common fibrotic ILD is idiopathic pulmonary fibrosis (IPF) (1). Besides ILDs of a granulomatous origin (e.g., sarcoidosis) and those of a known cause (e.g., asbestosis), the classification for idiopathic interstitial pneumonias (IIPs) has been agreed 10 years ago and likely needs to be reviewed as new data emerges on the interplay between genetic and environmental factors (2).

The terminology to refer to cases of ILD with a familial predisposition can be confusing. While initial reports used the term “familial IPF,” this would not account for the heterogeneity of ILD diagnoses in affected family members (not all relatives being diagnosed with IPF) (3). A commonly used term is familial pulmonary fibrosis (FPF) which expands the definition to cases of fibrotic ILDs within the same family. In addition, familial interstitial pneumonia (FIP) applies to families in which two or more cases of IIPs are diagnosed; e.g. index case (also called proband) having IPF, and a sibling diagnosed with fibrotic non-specific interstitial pneumonia (NSIP) (3). Finally, we may even use “familial ILD” to expand the definition to pedigrees where some relatives have fibrotic IIPs, while others are diagnosed with non-IIP ILDs or non-fibrotic ILDs, as we have encountered such families in our practice.

## 2. Genomic features of familial pulmonary fibrosis

### 2.1. Telomere shortening

Telomeres consist of non-coding DNA (*TTAGGG*) and associated proteins thought to maintain genomic integrity during repeated cell divisions. Critical TS can lead to diverse clinical syndromes (4). *Dyskeratosis congenita* is a prototype of a TS condition, which is described as a triad of oral leukoplakia, reticular pigmentation of the neck/upper chest and dysplastic nails (5). It can be associated with other features which may also be found in telomere-related FPF, such as bone marrow failure, myelodysplasia, and leukemia (6). TS syndrome can also be associated with immunodeficiency, neurological or retinal conditions (5, 7).

Telomere length decreases with age even in the absence of fibrotic lung pathology. However, this appears to be more pronounced in ILD patients (8). Those carrying a pathogenic variant (also called mutation) in a telomere-related gene (TRG) generally have shorter telomeres (<25th percentile) compared to age matched controls; while only 50% of ILD patients >60 years old and *TERT*, *TERC* or *RTEL1* mutations had a telomere length > 10th percentile (9). Short telomere length is associated with worse clinical outcomes in fibrotic ILD (10–12) and poor tolerance to immunosuppressive treatments (13, 14).

Telomere abnormalities, including TS, have also been identified in sporadic cases of IPF and other forms of pulmonary fibrosis (PF) (10, 15, 16).

### 2.2. Pathogenic variants in telomere-related genes

TRGs include *TERT, TERC, RTEL1, PARN, DKC1, TINF2, NOP10, NHP2, ACD, NAF1, ZCCHC8, RPA, POT1* (5). *TERT* (telomerase reverse transcriptase) and *TERC* (telomerase RNA component) are components of the telomere complex which is a group of proteins and RNA that drive the addition of telomeric repeats (*TTAGGG*) to the ends of chromosomes and were the first TRGs identified in FPF (4).

The mean age of ILD diagnosis in patients with TRG mutation is approximately 58 years (16). Clinical features include a usual interstitial pneumonia (UIP) radiological pattern on chest CT which can be seen in up to 54–74% of ILD cases with TRG mutations although other radiological patterns can be present. Radiological and histological patterns can vary even within the same family (16–18). *Hematological abnormalities*, such as anemia (17–27%), macrocytosis (24–41%) or thrombocytopenia (8–54%) may be associated to ILD (16, 17). Patients with *DKC1*, *TINF2* and *TERC* mutations are more likely to have hematological involvement compared to those with *TERT*, *PARN* or *RTEL1* mutations (16). Asymptomatic *liver function abnormalities* (i.e., increased liver transaminases) can also be found in 5–27% TRG mutation patients and *early hair greying* is noted in 15–40% (11, 19).

### 2.3. Pathogenic variants in surfactant-related genes and MUC5B polymorphism

Surfactant-related genes (SRGs) include *SFTPC* which has an autosomal dominant mode of inheritance with frequent *de novo* mutations, but low frequency in FPF cases (<5%) (5). *ABCA3* is mostly associated with respiratory failure in the newborn but severe adult ILD has been reported. *NKX2.1* encodes the protein TTF1 which regulates surfactant protein as well as *ABCA3* gene transcription. While NKX2.1 mutation mostly causes neonatal disease it can result in an atypical UIP pattern in adults (5, 20–23). In childhood, SRG mutations are the most common, whereas in adults, TRG pathogenic variants are more common (5, 24).

The single-nucleotide polymorphism rs35705950 in the promoter of the gene encoding mucin 5B (MUC5B) was shown to be associated with both FIP and sporadic IPF (25). It showed a significant signal in large genome-wide association studies and has been associated with various fibrotic ILDs (10, 26–29). However, given the high prevalence of the *MUC5B* rs35705950 minor allele promoter variant and low penetrance, genotyping of the variant is not currently part of FPF gene investigations in most countries (5). Fortunately in the UK, *MUC5B* is listed in the FPF panel, as well as SRGs and TRGs, as part of the National Genomic Test Directory for rare and inherited disease (30).

In FPF, there is overall a 50% chance of inheriting a deleterious allele in first-degree relatives (autosomal dominant) (5). Penetrance is generally incomplete, higher for SRGs (24) compared to TRGs, and expressivity is variable. The absence of TRG mutations in a family member of an index case does not exclude risk of disease due to other telomere abnormalities. The average age of ILD onset is lower in SRG compared to TRG mutation carriers. Pulmonary-only disease is more common in patients with SRG versus TRG pathogenic variants (5).

### 2.4. Senescence – the aging lung

Cellular senescence is characterized by a state of cell cycle arrest (31). PF is considered a disease of senescence (aging) with fibrogenesis requiring an interplay between genetic predisposition and repeated exogenous insults (i.e., environmental). Animal models have confirmed that increasing age and having mutations in TRGs (e.g., *TERT*) favor the development of fibrosis following infectious or noxious challenge. Immune processes can also be affected by cellular senescence (such as T cell dysfunction and shifting to Th17 responses leading to immune dysregulation and propensity to autoimmune responses) and environmental exposures can accelerate this process (31, 32). Genomic instability due to *TERT* or *ABCA3* mutations and fibrogenic exposures can lead to lung epithelial cell phenotypes that correspond to age-induced senescence (33).

There is evidence that acute exacerbations of IPF may correlate to air pollution, possibly mediated by senescence in inflammatory cells (34, 35). Fibroblast senescence can be triggered directly by environmental stress as well as indirectly by neighboring senescent cells altering the cellular microenvironment (36, 37). The window when environmental challenge occurs, as well as the dose and repeated exposure, play a role in senescence and fibrotic responses (33, 38).

## 3. Air pollution and environmental impact on genetic predisposition

Air pollution may trigger alterations on the airway mucosal surface by overwhelming ciliary and macrophage clearance mechanisms leading to oxidative stress and transporting toxic metals and particulates into the blood stream (39). It has been associated with increased incidence and worse outcomes in IPF, but large cohorts are required to validate these findings (40). Tobacco smoke, ozone exposures leading to oxidative stress by generating reactive oxygen species, lead and nitrosamines have also been linked to TS (41). Some of these effects may occur prenatally, e.g., in mothers exposed to high PM_2.5_ (particulate matter with an aerodynamic diameter ≤ 2.5 μm) air pollution (42). The onset of acute exacerbations of IPF was linked to air pollution, notably increased ozone concentrations (35).

Long term cumulative exposure to air pollution can increase the incidence of IPF, which is additive to genetic predisposition (43). Single gene mutations are thought to have relatively small effects, hence polygenic risk scores (PRS) have been proposed (44). Data from a large epidemiological study of 433,738 participants seems to support a role for air pollution in the pathogenesis of IPF in individuals stratified by PRSs. Those with a high PRS (13 single nucleotide polymorphisms) exposed to airborne pollutants had the highest risk of IPF incidence compared to low air pollution and low PRS in terms of adjusted hazard ratios (95% CI): for NO_2_ = 3.94 (2.77–5.6), NO_x_ = 3.08 (2.21–4.27), PM_2.5_ = 3.65 (2.6–5.13), and PM_10_ = 3.23 (2.32–4.5) (43).

Exposure of human bronchial epithelial cells to PM_2.5_ can lead to dose-dependent epigenomic modifications, altered telomerase activity and TS (45). IPF patients exhibit epigenomic modifications including widespread DNA methylation alterations in lung tissue which seem to occur near genes linked to PF (such as *TOLLIP*, *NOTCH1*, *FBXO32*) (46, 47). Resistance to fibroblast was linked to histone modifications, and fibroblast proliferation appears to be regulated by noncoding RNAs (40). Increased road traffic-derived PM_10_ exposure as measured by air pollution monitors in Beijing was associated with decreased histone H3 methylation (48). Increased PM_10_ exposure has also been associated with accelerated forced vital capacity (FVC) decline in IPF patients (34). Increased levels of both PM_2.5_ and PM_10_ were associated with higher IPF mortality (35).

## 4. Clinical considerations in FPF

### 4.1. Disease progression in familial versus sporadic fibrotic ILDs

Progressive pulmonary fibrosis (PPF) is the dominant phenotype seen in familial cases. There is evidence to suggest that FPF is worse, faster progressing and has higher mortality than sporadic fibrotic ILD, although data is incomplete and at times contradictory (5). In a small study, a 9.9% annual rate of FVC decline was seen in familial IPF patients compared to 4.9% in those with sporadic IPF (not statistically significant, *p* = 0.12) (49). The average annual rate of FVC decline amongst 115 ILD patients with TRG mutations (46% of whom had IPF) was found to be 300 ml (regardless of gene involved) (16). The mean survival from diagnosis in FPF appears to be between 2.4 and 7.3 years (18, 50). In a cohort of 1,262 ILD patients, survival was found to be worse in FPF compared to sporadic PF, both for IPF cases (HR for death or transplant of 1.8 [95% CI, 1.37–2.37]) and non-IPF ILD (HR for death or transplant of 2.08 [CI: 1.46–2.96]) (51). Regardless of the ILD diagnostic label, there was no difference in median survival comparing familial IPF to familial non-IPF (16).

### 4.2. Dedicated familial interstitial pneumonia clinics

As genomic tests can be expensive or only reimbursed when certain criteria are met, it is recommended to pre-screen patients based on family history and clinical features of TS prior to testing (Tables 1, 2) (5, 52). In our opinion, the easiest way to organize testing is by running dedicated FIP clinics. This allows to pool together patients with rare ILDs, screen relatives, offer personalized medicine and identify associated conditions (e.g., hematological diseases). More time for explanations can be given by a trained healthcare provider leading to a better patient experience. Genetic and psychological counselling, specialist ILD nursing and research can also be integrated within the FIP clinic structure.

**Table 1:** Clinical features of short telomere syndrome which can be elicited during consultations of patients affected by familial pulmonary fibrosis and their relatives.

Clinical features of telomere shortening: ONE or more of these features
1.Fibrotic ILD diagnosis before age 50
2.One or more relatives with ILD or known mutations in SRGs or TRGs
3.Other relevant personal or family history: (a)*Hematological abnormalities*: macrocytosis, neutropenia, lymphopenia, thrombocytopenia, myelodysplasia, acute leukemia, bone marrow failure (b)*Hepatic abnormalities*: unexplained elevated liver enzymes, portal hypertension, hepato-pulmonary syndrome, liver cirrhosis (c)Autoimmune abnormalities or connective-tissue disease features (d)Significant and premature hair greying, or developing streaks of grey hair (age < 30 years) (e)Early unexplained menopause (age < 45 years) (f)Frequent malignancy in the family (g)Dyskeratosis congenita or aplastic anemia

Features are adapted from the European Respiratory Society statement on familial pulmonary fibrosis (5) and Molina-Molina et al. (52).

**Table 2:** Eligibility for genomic testing for familial pulmonary fibrosis as per the latest version of UK National Genomic Test Directory for rare and inherited disease (53).

UK NHS National Genomic Test Directory – Testing Criteria for Rare and Inherited Disease, version 5.1, May 2023
R421 Pulmonary Fibrosis Familial testing criteria: ILD and ONE of the following:
ILD, no identifiable cause or association, and age < 50 years.
Family history of ILD regardless of identifiable cause or association.
For suspected telomerase complex mutations, testing to be considered in the absence of 1. and 2. above if one or more of the following are present in addition to ILD: Unexplained hematological abnormalities including macrocytosis, anemia, thrombocytopenia, leukopenia and/or lymphopenia; premature greying, Or unexplained liver function abnormalities. Consideration of lung transplantation.

Appropriate counselling and a tactful approach are necessary because genomic testing can be seen as opening a “Pandora’s box.” It often leads to major changes in patients’ lives and those of their relatives. In our experience with the Manchester FIP clinic, most patients are extremely interested in genomic tests if the wider context is clearly explained, and a pathway exists to deal with possible results. Importantly, testing provides an answer to the question “Why did I get ILD?” which leads to better acceptance of the ILD diagnosis and treatment requirements. Many patients are also very interested in research projects.

### 4.3. Impact on ILD multidisciplinary team (MDT) decisions

In our experience, familial and sporadic cases with telomere dysfunction frequently have atypical ILD presentations, and this can delay referral to an ILD specialist center, accurate diagnosis, and timely treatment.

As FPF cases may progress faster than sporadic ones, more frequent follow-up with lung function monitoring and early lung transplant referral should be considered as part of the MDT discussion, ideally involving transplant specialists and genetic counsellors. Transplant outcomes between FPF and sporadic PF were comparable (54, 55), therefore knowledge of a familial aggregation, TS or identified mutation should not automatically be considered contraindications for lung transplantation (54). Special considerations should be given to personalizing immunosuppressive regimens and the post-transplant follow-up as patients with telomere dysfunction (TS and/or TRG mutation) may be at increased risk of complications (13, 14, 56, 57).

Antifibrotics are just as useful in FPF as in sporadic fibrotic ILDs (IPF and PPF). Immunosuppression can increase the risk of complications and efficacy may be limited in patients who require such treatments (e.g., to treat fibrotic hypersensitivity pneumonitis) (14). It would be reasonable to encourage smoking cessation and avoidance of other inhalational exposures.

### 4.4. Interstitial lung abnormalities and early diagnosis

Interstitial lung abnormalities (ILAs) on chest CT scanning are a common finding in relatives of FPF patients (5). They are defined as incidental findings of nondependent abnormalities which affect >5% of any lung zone (upper, middle, lower), where ILD was not previously suspected. Respiratory symptoms may be present, and this may indicate early ILD. Three subtypes of ILAs have been described: non-subpleural nonfibrotic, subpleural nonfibrotic, subpleural fibrotic (58, 59).

Previous studies have demonstrated a prevalence of ILAs on lung cancer screening of 4–20% (60–63). Recent data from Manchester found a prevalence of ILAs on low-dose chest CT scanning of 3.9%, and 40.7% of these individuals with ILAs were subsequently diagnosed with ILD within 5 years (64).

Interstitial lung abnormalities are associated with radiological progression and mortality (64). Having traction bronchiectasis or bronchiolectasis is an important radiological risk factor to predict adverse outcomes (65, 66).

### 4.5. How to screen relatives of FPF patients?

There is no clear pathway or optimal age for screening relatives of FPF patients. As there is a 1–3-year delay between symptom onset and diagnosis for FPF patients, this represents an opportunity for early diagnosis (67).

It is recommended that all first-degree relatives of FPF patients should have a screening chest high-resolution CT (HRCT) scan especially if they have non-resolving respiratory symptoms (chronic cough and dyspnea) (5). The prevalence of interstitial lung changes on CT screening of first-degree relatives of FPF patients has been reported as 14–25% (5). Among first-degree relatives of FPF without overt ILD at screening, 19.4% developed extensive HRCT abnormalities or clinical ILD at 5 years. Also, 63.3% of patients with limited ILAs at enrolment experienced progression compared to only 6.1% of patients with normal HRCT at baseline (68).

Lung function is important for screening and follow-up, but simple spirometry may not identify early cases since FVC < 80% predicted in asymptomatic relatives is rare. However, relatives with ILAs have a lower diffusing capacity of the lung for carbon monoxide (DLCO) than those with normal HRCT (68, 69). Annual lung function testing may identify trends of decline. The role of home spirometry monitoring has not been explored in this population but may hold promise for the future.

Additionally, due to the high prevalence of other TS features, a full blood count (FBC) and liver function testing should also be performed in first-degree relatives (5).

Certain relatives may be more open to screening, while others would prefer to wait and clinicians should abide by their preferences, to not cause undue anxiety. Eligible relatives may also be offered genomic testing, usually via local/regional Clinical Genetics services who follow-up families of the proband.

To date, there is no consensus on the frequency and duration of clinical, functional, and radiological evaluations of first-degree relatives of FPF patients. Management could be customized through a risk assessment, considering resources available in each country/national health system.

**Figure 1:**
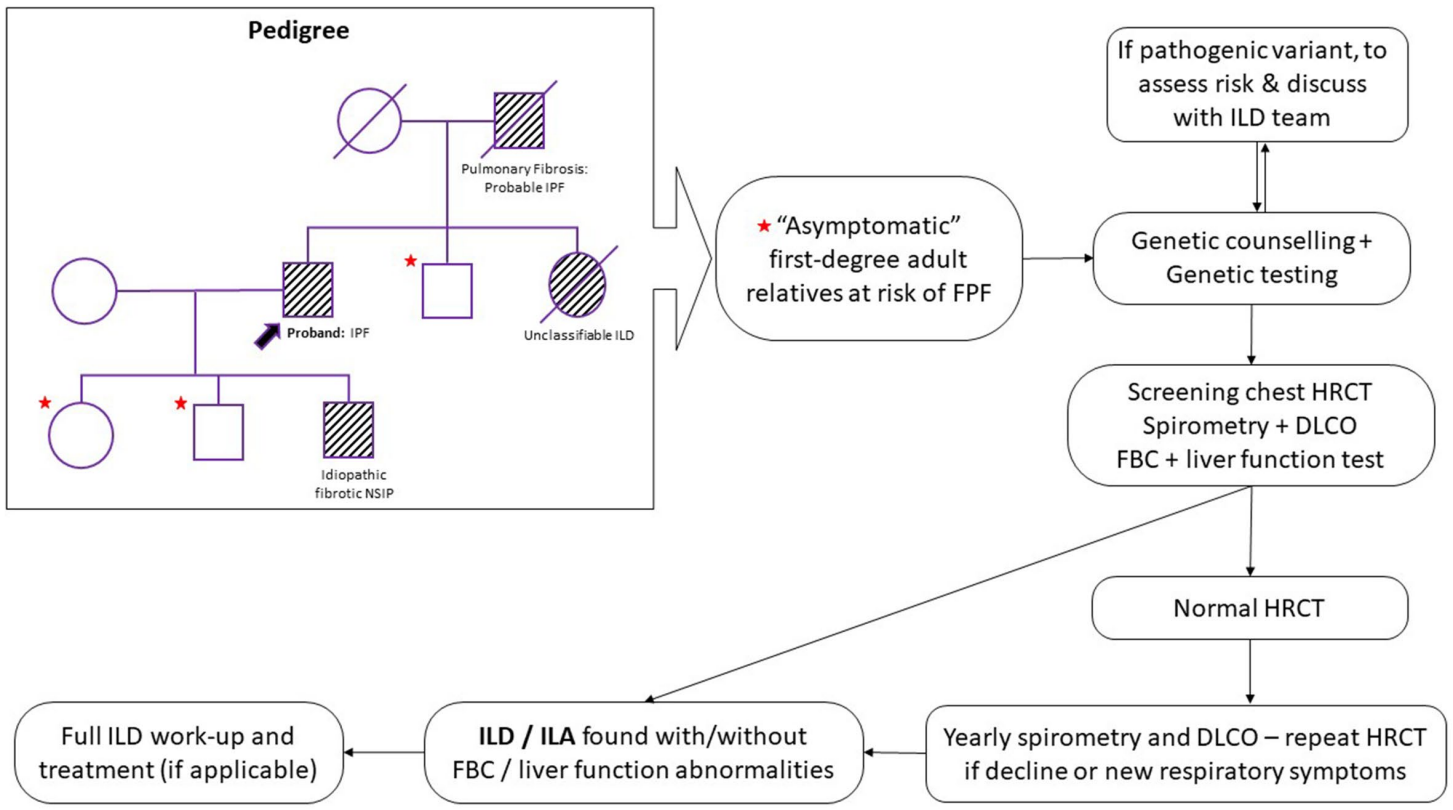
Proposed screening and monitoring pathway for first-degree relatives of familial pulmonary fibrosis (FPF) patients. HRCT, high resolution computed tomography; DLCO, diffusing capacity of the lung for carbon monoxide; FBC, full blood count; ILD, interstitial lung disease; ILA, interstitial lung abnormalities; IPF, idiopathic pulmonary fibrosis; NSIP, non-specific interstitial pneumonia.

Figure 1 outlines a potential screening strategy for relatives of FPF patients.

## 5. Disparities and barriers to genomic testing

Although patients with PF were found to have disproportionately short leukocyte telomere length (LTL), there was very little genetic variability in cohorts (to account for ethnic and socio-economic differences around the world). LTL decreased with age, more so in PF patients compared to controls (*R* = −0.28, *p* < 0.0001). It appears that sex-adjusted LTL below median is uniformly associated with chronological age and increased risk of mortality in all racial groups although in the Asian population it was not statistically significant (White = HR 2.21, [95%CI: 1.79–2.72], Black = HR 2.22 [1.05–4.66], Hispanic = HR 3.40 [1.88–6.14], Asian = HR 2.11 [0.55–8.23]) (8). Fibrotic lung disease seems to occur several years earlier in African American compared to White patients (70). Patients of Black ethnicity are less likely than those of White ethnicity to have lung transplantation and have worse outcomes even when controlling for confounding factors, socio-economic status (as per the area deprivation index – ADI), donor cause of death, blood type nor HLA mismatch (71, 72). Further research to determine if ethnic differences or socio-economic disparities drive outcomes in FPF are needed (73).

The availability of genomic testing, telomere length measurement and whole genome sequencing varies greatly around the world, making it difficult to standardize global recommendations. At the time of this writing, a 25-gene panel test for FPF is available in the UK, but telomere length measurement is not reimbursed by the health service (30). Local laws and practice regarding genetic counselling can also vary greatly around the world (74).

## 6. Conclusion

In our opinion, actively eliciting a family history of ILD and other rare conditions, as well as asking about TS features (Tables 1, 2) could be a cost-effective way of identifying relatives at risk of developing ILD. We hypothesize that FPF is more frequent than previously thought, but patients are rarely asked specifically about it during routine ILD consultations. We believe that dedicated FIP clinics can improve care through personalized medicine, early screening and diagnosis in “asymptomatic” relatives, genomic testing, rapid access to antifibrotic medication and early lung transplant referral. We also feel that environmental exposures should be elicited due to the additive risk in FPF (43), and this can be done by using standardized questionnaires (75).

## Data availability statement

The original contributions presented in the study are included in the article/supplementary material, further inquiries can be directed to the corresponding author.

## Author contributions

SS and PR-O contributed equally to the conceptualization and literature search. JC contributed to summarizing the key points from the references. SS and PR-O structured the manuscript. SS, JC, and PR-O provided comments and contributed equally to finalizing the draft manuscript for submission. All authors contributed to the article and approved the submitted version.

## Conflict of interest

The authors declare that the research was conducted in the absence of any commercial or financial relationships that could be construed as a potential conflict of interest.

## Publisher’s note

All claims expressed in this article are solely those of the authors and do not necessarily represent those of their affiliated organizations, or those of the publisher, the editors and the reviewers. Any product that may be evaluated in this article, or claim that may be made by its manufacturer, is not guaranteed or endorsed by the publisher.
